# Wide-Open: Accelerating public data release by automating detection of overdue datasets

**DOI:** 10.1371/journal.pbio.2002477

**Published:** 2017-06-08

**Authors:** Maxim Grechkin, Hoifung Poon, Bill Howe

**Affiliations:** 1 Paul G. Allen School of Computer Science & Engineering, University of Washington, Seattle, Washington, United States of America; 2 Microsoft Research, Redmond, Washington, United States of America; 3 Information School, University of Washington, Seattle, Washington, United States of America

## Abstract

Open data is a vital pillar of open science and a key enabler for reproducibility, data reuse, and novel discoveries. Enforcement of open-data policies, however, largely relies on manual efforts, which invariably lag behind the increasingly automated generation of biological data. To address this problem, we developed a general approach to automatically identify datasets overdue for public release by applying text mining to identify dataset references in published articles and parse query results from repositories to determine if the datasets remain private. We demonstrate the effectiveness of this approach on 2 popular National Center for Biotechnology Information (NCBI) repositories: Gene Expression Omnibus (GEO) and Sequence Read Archive (SRA). Our Wide-Open system identified a large number of overdue datasets, which spurred administrators to respond directly by releasing 400 datasets in one week.

## Letter

**Fig 1:**
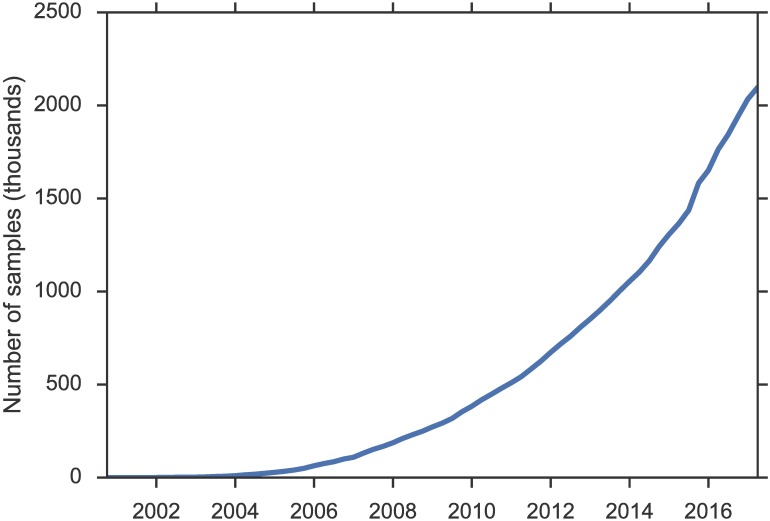
Number of samples in the National Center for Biotechnology Information (NCBI) Gene Expression Omnibus (GEO). Data underlying the figure are available as S1 Data.

Advances in sequencing and other biotechnologies have led to an explosion of biological data. Fig 1 shows the remarkable growth in the number of gene expression samples in the National Center for Biotechnology Information (NCBI) Gene Expression Omnibus (GEO) repository [1]. As of February 2017, GEO contains 80,985 public datasets and 2,097,543 samples, spanning hundreds of tissue types in thousands of organisms. Making such a wealth of data publicly available not only facilitates replication but also generates new opportunities for discovery by jointly analyzing multiple datasets [2].

Consequently, journals and repositories have increasingly embraced open-data policies. *PLOS* journals require authors to "make all data underlying the findings described in their manuscript fully available without restriction" [3]. GEO requests that authors should inform them "as soon as your manuscript is published so that we can release your records and link them with PubMed" (https://www.ncbi.nlm.nih.gov/geo/info/faq.html#holduntilpublished). Enforcing such policies, however, largely relies on manual efforts. Authors often forget to notify repositories when their papers get published. Repositories such as GEO resort to periodically checking private datasets to determine if they should be released and calling upon users to notify them of overdue ones. Still, the lag between the date the paper is published and the date the data are released is significant and appears to grow over time.

To help address the opportunity cost of this "hidden data," and to reduce the burden of manually keeping track of the release process for authors and repository administrators, we developed Wide-Open, a general approach that applies text mining to automatically detect overdue datasets in a public repository.

Wide-Open first scans PubMed articles for dataset unique identifiers (UIDs) by using regular expressions. It then determines the validity of each candidate UID, and whether the corresponding datasets have been released. To determine if the dataset has been released, Wide-Open calls the repository's web application programming interface (API) for accessing datasets and searches for signature textual patterns in the query result. When there exists a database that indexes many publicly released datasets, Wide-Open will first check the UIDs by using the database to minimize unnecessary web API calls.

To evaluate the effectiveness of this approach, we applied it to two popular NCBI repositories: GEO and Sequence Read Archive (SRA). To scan PubMed text for accession numbers, Wide-Open uses the regular expression GSE[0–9]+ for GEO, and SRX[0–9]+ for SRA. For each candidate accession number, Wide-Open first checks GEOmetadb [4] for GEO, and SRAdb [5] for SRA. A hit means that the dataset has been released. If not, Wide-Open calls the web APIs for GEO (https://www.ncbi.nlm.nih.gov/geo/query/acc.cgi?acc=<accession>) and SRA (https://www.ncbi.nlm.nih.gov/sra/?term=<accession>). The resulting page will then be parsed to determine if the accession number is valid and, if so, whether the dataset is public or private. In the latter case, the dataset remains private after being cited in a published article, which means that it is most likely to be overdue.

Specifically, for GEO, Wide-Open looks for strings such as "Could not find a public or private accession," which signifies an invalid accession number, as well as strings such as "is currently private," which signifies that the dataset is private. For SRA, the process is similar. The details can be found in our open-sourced code.

**Fig 2:**
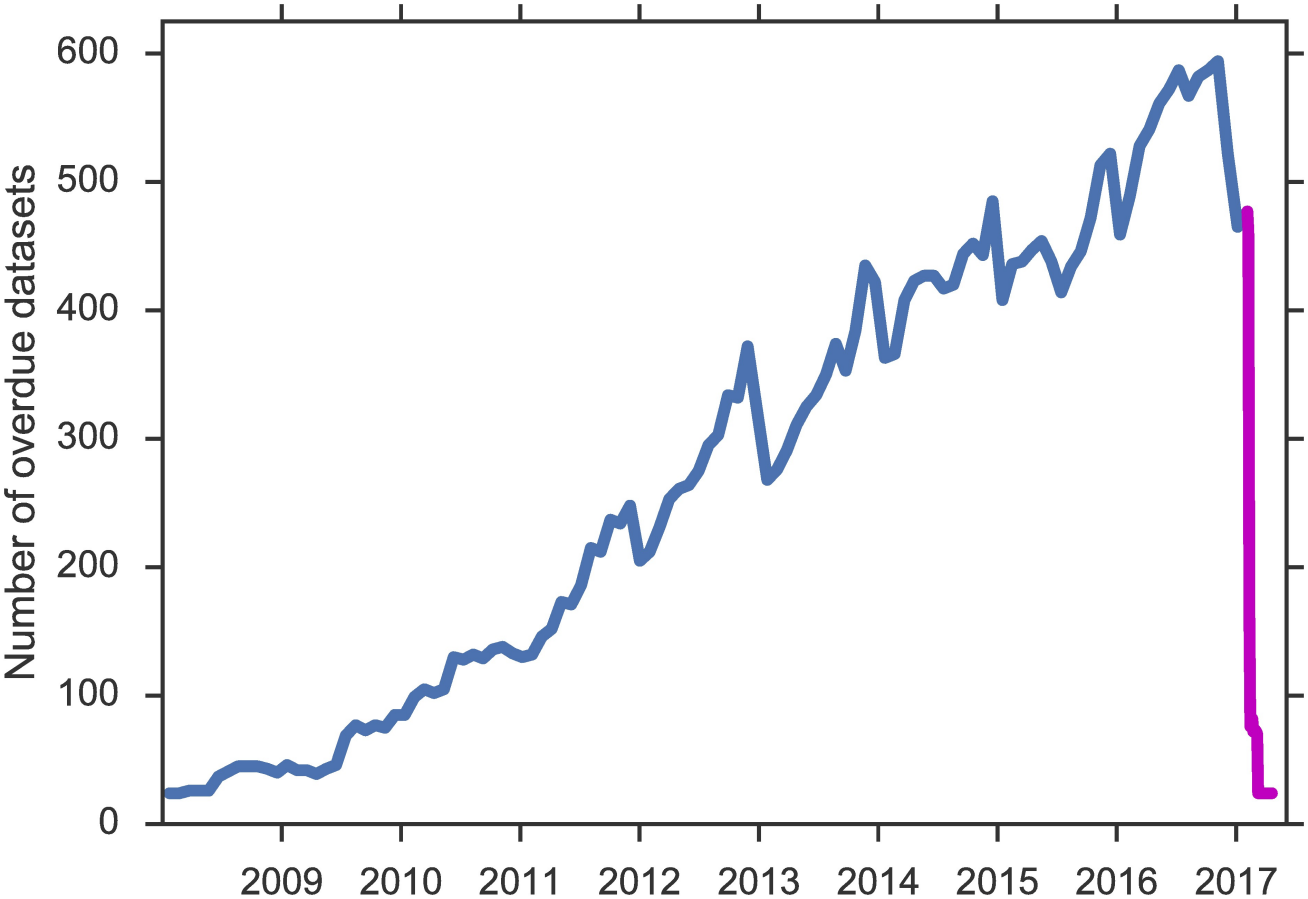
Number of Gene Expression Omnibus (GEO) datasets overdue for release over time, as detected by Wide-Open. Prior to this submission, we notified GEO of the standing list, which led to the dramatic drop of overdue datasets (magenta portion), with 400 datasets released within the first week. Data underlying the figure are available as S2 Data.

Wide-Open identified a large number of overdue datasets in GEO and SRA. Fig 2 shows the number of overdue GEO datasets over time. For each time point, we show the number of datasets referenced in prior publications but not yet released at the time of publishing. Notwithstanding some fluctuation, the number has been steadily rising since the advent of next-generation sequencing. The oldest paper that references an overdue dataset was published in 2010. Prior to this submission, we notified GEO of the overdue datasets that Wide-Open had identified. We received a prompt acknowledegement and noticed a dramatic drop in the number shortly after our exchange (the magenta portion; approximately 400 datasets were released within the first week). We applaud the quick action by GEO and take this response as a promising sign that an automatic monitoring system like Wide-Open could help accelerate the release process. Out of the 473 datasets identified by Wide-Open in February 2017, 455 have been released by GEO since. Of the remaining 18 candidates, only one is a true precision error (the accession number candidate GSE17200 actually refers to a soil name). Among the other 17 cases, 14 were identified due to typos by the authors who cited a wrong accession number, while the remaining 3 were legitimate datasets that could not be released either due to incomplete submission or privacy issues. In other words, Wide-Open attained a precision of 97%, even with author errors considered.

As of March 2017, Wide-Open has identified 84 overdue SRA datasetsas. Next, we plan to contact SRA and work with them on verification and release of these datasets as well.

**Fig 3:**
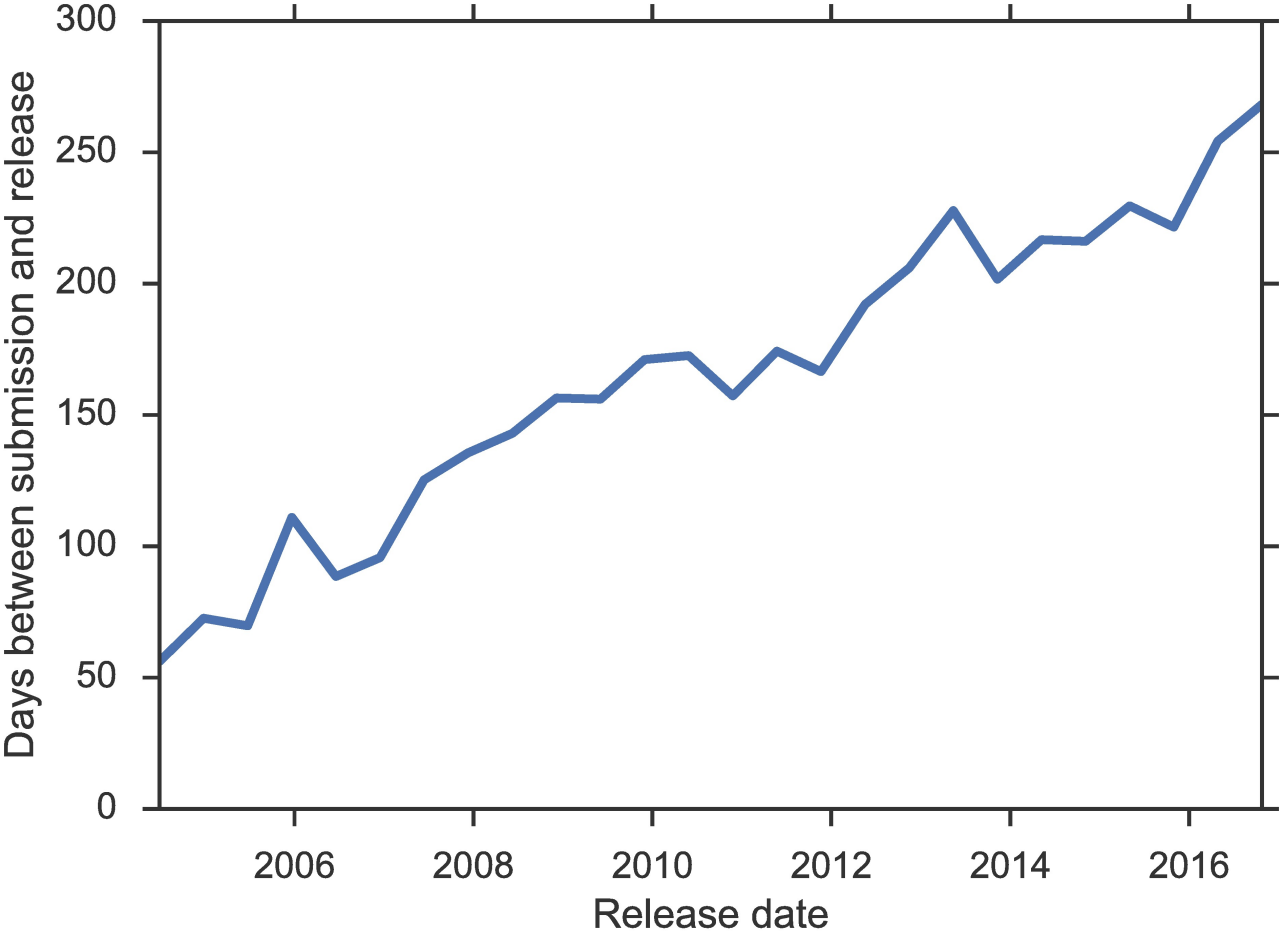
Average delay from submission to release in the Gene Expression Omnibus (GEO). Data underlying the figure are available as S3 Data.

The time lag between submission and release has also steadily risen (Fig 3). GEO datasets that became public in 2006 took an average of 87 days from submission to release, whereas in 2016, the average delay was over 8 months. GSE2436 was submitted to GEO in March 2005 and was not made public until November 2016, an 11-year wait. While longer reviewing cycles might explain part of this increase [6], it seems clear that the rapid growth in the number of datasets would tax the manual release process and ultimately make it unsustainable.

While the initial progress is promising, much remains to be done. We need full-text access to identify published datasets, which limits our current monitoring to the open access subset of PubMed Central (PMC). As of February 2017, this subset contains about 1.5 million papers, which is a small subset of PMC (4.2 million) and a fraction of PubMed (26 million). There are various ways to substantially increase the number of full-text articles for monitoring thanks to the open-access movement championed by this journal and others. Publishers are increasingly open to granting text-mining licenses (e.g., http://text.soe.ucsc.edu/progress.html). Through our collaborators, we begin to have access to many more full-text articles on which we plan to run Wide-Open next. The number of private datasets is rather large. For example, GEO currently has over 10,000 datasets that remain private. We expect that many more overdue datasets could be identified with access to additional full-text articles.

Wide-Open is available under an open source license at GitHub (https://github.com/wideopen/datawatch). We will host a service to keep monitoring publications and identifying overdue datasets (https://wideopen.github.io/datawatch/). We also plan to extend *Wide-Open* to cover more repositories and implement more fine-grained audits (e.g., making sure that the released dataset contains at least as many samples as reported in the article). Extending Wide-Open to a new repository consists of 3 simple tasks: creating regular expressions for dataset identifiers, identifying the web API for dataset access, and adapting the query-result parser to distinguish between invalid UIDs, datasets that have been released, and datasets that remain private. We envision Wide-Open as a collective project to engage the research community and help advance the open-data movement.

## Supporting information

S1 Data Number of samples in GEO. Obtained from https://www.ncbi.nlm.nih.gov/geo/summary/summary.cgi?type=history.
(CSV)

S2 Data Number of overdue datasets in GEO. Computed using Wide-Open extracted references, GEOmetadb and queries against GEO web interface as described in the paper.
(CSV)

S3 Data Average lag between submission to GEO and release of the dataset. Computed using data from GEOmetadb.
(CSV)

S4 Data sqlite data: WideOpen database of extracted references. (XZ)

## Abbreviations

GEO: Gene Expression Omnibus
NCBI: National Center for Biotechnology Information
PMC: PubMed Central
SRA: Sequence Read Archive
UID: unique identifier
